# Marek’s disease virus infection induces widespread differential chromatin marks in inbred chicken lines

**DOI:** 10.1186/1471-2164-13-557

**Published:** 2012-10-16

**Authors:** Apratim Mitra, Juan Luo, Huanming Zhang, Kairong Cui, Keji Zhao, Jiuzhou Song

**Affiliations:** 1 Department of Animal & Avian Sciences, University of Maryland, College Park, MD, USA; 2 USDA, ARS, Avian Disease and Oncology Laboratory, East Lansing, MI, USA; 3 Laboratory of Molecular Immunology, National Heart, Lung and Blood Institute, National Institutes of Health, Bethesda, MD, USA

**Keywords:** Histone modifications, Thymus, Differential marks, Bivalent domain, Chromatin signature, Marek’s disease

## Abstract

**Background:**

Marek’s disease (MD) is a neoplastic disease in chickens caused by the MD virus (MDV). Successful vaccine development against MD has resulted in increased virulence of MDV and the understanding of genetic resistance to the disease is, therefore, crucial to long-term control strategies. Also, epigenetic factors are believed to be one of the major determinants of disease response.

**Results:**

Here, we carried out comprehensive analyses of the epigenetic landscape induced by MDV, utilizing genome-wide histone H3 lysine 4 and lysine 27 trimethylation maps from chicken lines with varying resistance to MD. Differential chromatin marks were observed on genes previously implicated in the disease such as *MX1* and *CTLA-4* and also on genes reported in other cancers including *IGF2BP1* and *GAL.* We detected bivalent domains on immune-related transcriptional regulators *BCL6, CITED2* and *EGR1*, which underwent dynamic changes in both lines as a result of MDV infection. In addition, putative roles for *GAL* in the mechanism of MD progression were revealed.

**Conclusion:**

Our results confirm the presence of widespread epigenetic differences induced by MD in chicken lines with different levels of genetic resistance. A majority of observed epigenetic changes were indicative of increased levels of viral infection in the susceptible line symptomatic of lowered immunocompetence in these birds caused by early cytolytic infection. The *GAL* system that has known anti-proliferative effects in other cancers is also revealed to be potentially involved in MD progression. Our study provides further insight into the mechanisms of MD progression while revealing a complex landscape of epigenetic regulatory mechanisms that varies depending on host factors.

## Background

Rapid advances in epigenetics have led to the discovery of complex mechanisms of gene regulation involving phenomena such as DNA methylation and chromatin modifications. Methylation of particular histone residues has been found to correlate with specific and often opposing cellular functions, e.g. trimethylation of histone H3 lysine 4 (H3K4me3) is associated with transcriptional start sites (TSSs) of active genes while trimethylation of histone H3 lysine 27 (H3K27me3) is found to mark broad genomic regions for repression. Recent studies have also suggested that characteristic combinations of histone modifications or ‘chromatin states’ define functional elements of the genome and determine their contribution to transcriptional regulation [1-3]. Moreover, the epigenetic state of host genes have been observed to be affected by viral infection leading to tumors in humans [4-6]. Thus, epigenetics constitute a dynamic regulatory framework linking genotypes with environmental factors that could play a major role in differential disease responses among individuals having high genetic similarity.

Marek’s disease (MD) is a highly contagious disease caused by an oncogenic α-herpesvirus MD virus (MDV) and characterized by T-cell lymphomas in chickens [7]. Major losses to the poultry industry as a result of MD have largely been averted due to the success of various vaccination strategies which, remarkably, is also the first instance of the successful control of a natural cancer-causing agent using vaccines [8-10]. However, the virulence of the virus may have progressively increased as a consequence of vaccine development [11-13]. Several reported instances of vaccine breaks or reduced efficacy of vaccination, therefore, underlines the importance of investigating resistance to the disease as a long-term strategy to control MDV.

Natural resistance to MDV can be divided into two categories: major histocompatibility complex (MHC)-associated resistance, wherein different MHC haplotypes at the B blood group locus confer varying levels of resistance and non-MHC associated resistance in which birds having the same MHC haplotype exhibit vastly different responses to MDV infection. Inbred lines 6_3_ and 7_2_ developed at the Avian Disease and Oncology Laboratory (ADOL, East Lansing, MI) that we used in this study, fall into the latter category. These lines share a high degree of genetic similarity but have divergent responses to MDV infection completely independent of the MHC. Several studies have attempted to pinpoint factors responsible for conferring resistance [14-16], but confounding factors, such as, tissue types, virus strains and ages of birds have made it difficult to find a consensus. Multiple risk elements are possibly at play in this complex disease, and increased resistance or susceptibility is likely to be produced by a combination of such factors. In this study, we take a closer look at epigenetic factors behind different responses to MD with a view to a deeper understanding of the broader genomic impact of MDV infection.

We utilized the above population of inbred chickens – line 6_3_ is highly resistant to MD, while line 7_2_ is highly susceptible – and compared the epigenetic effects of MD. Genome-wide maps of H3K4me3 and H3K27me3 in thymus tissues of birds from these chicken lines at the latent stage of MDV infection were generated. We carried out systematic analyses to find differential chromatin marks induced by MDV infection. We also investigated co-localization patterns of the two chromatin modifications to detect putative bivalent domains and the effect of MDV on such domains. The results of our study confirm that Marek’s disease has far-reaching effects on the epigenetic landscape of chicken lines with diverse responses to the virus and, thus, furthers our understanding of this complex disease.

## Results

### Genome-wide distribution of H3K4me3 and H3K27me3

We performed ChIP-Seq experiments on infected and uninfected birds from lines 6_3_ and 7_2_ to investigate the epigenetic effects of MDV infection. More than 76 million reads from eight samples were mapped to the chicken genome yielding 14418 and 24950 significantly enriched regions (SERs) for H3K4me3 and H3K27me3, respectively (Table 1). We further classified these regions as follows: Ubiquitous SERs were found in all samples and were likely due to similarities in the genetic background of the chickens. Line-specific SERs were present in only one line either before or after MDV infection, while condition-specific SERs appeared in both lines but only in individuals with the same infection status.

**Table 1:** Significantly enriched regions (SERs) and associated genes in each sample

		**H3K4me3**		**H3K27me3**	
	**Samples**	**SERs (%)**	**Genes**	**SERs (%)**	**Genes**
**Line-Specific**	**63I**	647 (4.5)	78	3477 (13.9)	615
	**63N**	594 (4.1)	71	2514 (10.1)	896
	**63I,63N**	924 (6.4)	190	577 (2.3)	150
	**72I**	105 (0.7)	16	1658 (6.6)	451
	**72N**	126 (0.9)	11	2506 (10)	346
	**72I,72N**	73 (0.5)	17	330 (1.3)	89
**Condition-specific**	**63I,72I**	97 (0.7)	35	2061 (8.3)	579
	**63N,72N**	47 (0.3)	9	66 (0.3)	22
**Ubiquitous**	**63I,63N,72I,72N**	10691 (74.2)	9475	5831 (23.4)	2942
	**Total**	**14418**	**10206**	**24950**	**7904**

63I: line 6_3_ infected, 63N: line 6_3_ control, 72I: line 7_2_ infected, 72N: line 7_2_ control.

Ubiquitous SERs formed the largest percentage of all enriched regions, accounting for 74.2% and 23.3% in H3K4me3 and H3K27me3 samples, respectively. In the case of H3K4me3, there were large differences in the number of specific SERs - more than 2000 line-specific SERs were found in line 6_3_, compared to about 300 in line 7_2_. Similarly, we found 50% more line-specific SERs of H3K27me3 in line 6_3_ (6568) compared to line 7_2_ (4494). However, upon closer examination, most of the line-specific and condition-specific SERs were revealed to have low read counts (Additional file 1: Figure S1) corresponding to regions of low enrichment.

**Figure 1:**
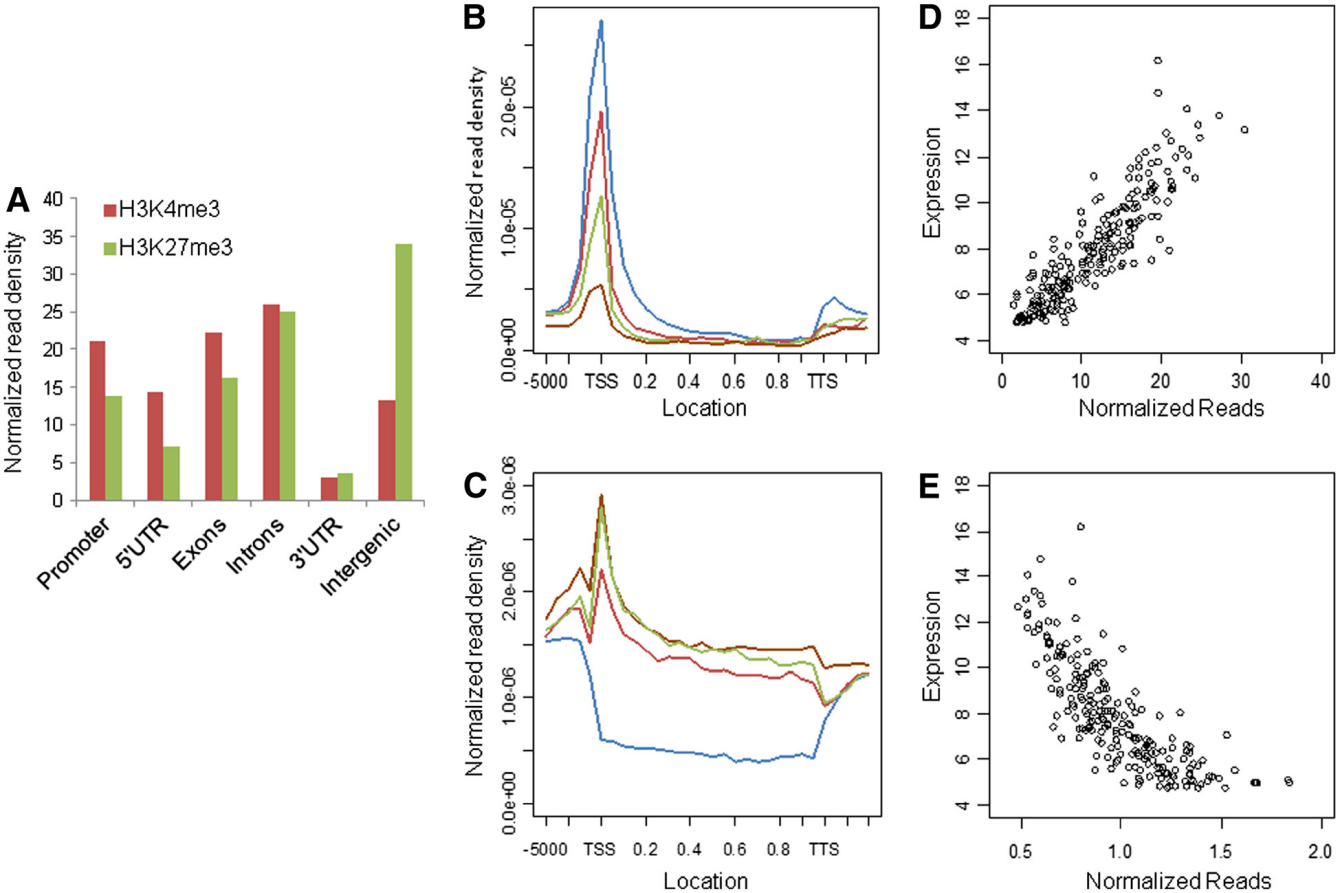
**Genomic distribution of SERs and relationship between histone marks and gene expression.** (**A**) Distribution of SERs over different genomic elements shows increased levels of H3K4me3 around the promoter region and exons while there are increased levels of H3K27me3 on intergenic regions. (**B**-**E**) Relationship between gene expression and histone marks in infected line 6_3_ birds. Plots of histone modifications around the gene body (**B**, **C**) in genes having high (blue), medium (red), low (green) and no activity (brown): H3K4me3 shows positive correlation with gene expression levels while H3K27me3 exhibits a negative relationship. A comparison of epigenetic marks and transcriptional levels (**D**, **E**) confirms the same. Similar trends were observed in other experimental groups (Additional file 1: Figures S3-5).

Genes were divided into five regions – promoter, 5’ untranslated region (UTR), exons, introns and 3’ UTR – and the distribution of SERs across these elements was probed (Figure 1A). We found a large number of intergenic regions marked by H3K27me3, consistent with high levels of this mark associated with areas of silent heterochromatin. In the case of H3K4me3, a larger proportion of SERs were found around the promoter, exons and the 5’ UTR while similar proportions of H3K4me3 and H3K27me3 SERs were present in introns and 3’ UTRs. A comparison of the genomic distributions of SERs in the different samples (Additional file 1: Figures S2A, B) showed a similar number of H3K4me3 SERs across the promoter, exons and the 5’ and 3’ UTRs of genes. Line 6_3_ contained a higher number of intronic and intergenic SERs as compared to line 7_2_ although this did not appear to change as a result of MDV infection. On the other hand, a greater number of H3K27me3 SERs were found in the infected samples although these levels were similar in the two different lines.

To analyze the relationship between histone modifications and gene expression, histone modification profiles surrounding the TSS and gene body were plotted for genes ranked by their expression level (Additional file 1: Figures 1B-E and S3-5). As expected, a strong positive correlation was observed between gene expression and H3K4me3 marks with a distinct peak around the TSS and little to no enrichment within the gene body. On the other hand, H3K27me3 showed negative correlation with gene expression with a peak near the TSS followed by a broad plateau across the gene body. However, the latter relationship was non-linear – genes with lower expression had similar levels of H3K27me3 marks and levels were markedly distinct only at higher expression levels (Figure 1C, E).

### Differential H3K4me3 marks on genes related to MD

We conducted a comprehensive analysis of genome-wide chromatin marks to find significant differences in MDV-induced responses in line 6_3_ and 7_2_. We used two sets of comparisons: First, to assess the influence of MDV infection within each line, we compared the infected and the non-infected samples from the same line. Secondly, the non-infected samples from the two lines were compared to detect line-specific effects. As a result of this analysis we found 179 differential H3K4me3 SERs and 1116 differential H3K27me3 SERs that mapped to 59 and 66 genes, respectively (Table 2). A majority of differential SERs were found in the comparison between non-infected samples of the two lines (Additional files 2 and 3) with several observed on genes that have been associated with MDV infection.

**Table 2:** Differential SERs identified in thymus

	**H3K4me3**		**H3K27me3**	
**Comparison**	Differential SERs*	**Genes**	Differential SERs*	**Genes**
**63I vs 63N**	9	4	42	1
**72I vs 72N**	30	13	5	0
**63N vs 72N**	148	46	1094	65
**Total**	**179**	**59**†	**1116**	**66**

*FDR < 0.4. † Some genes are shared between different comparisons. 63I: line 6_3_ infected, 63N: line 6_3_ control, 72I: line 7_2_ infected, 72N: line 7_2_ control.

**Figure 2:**
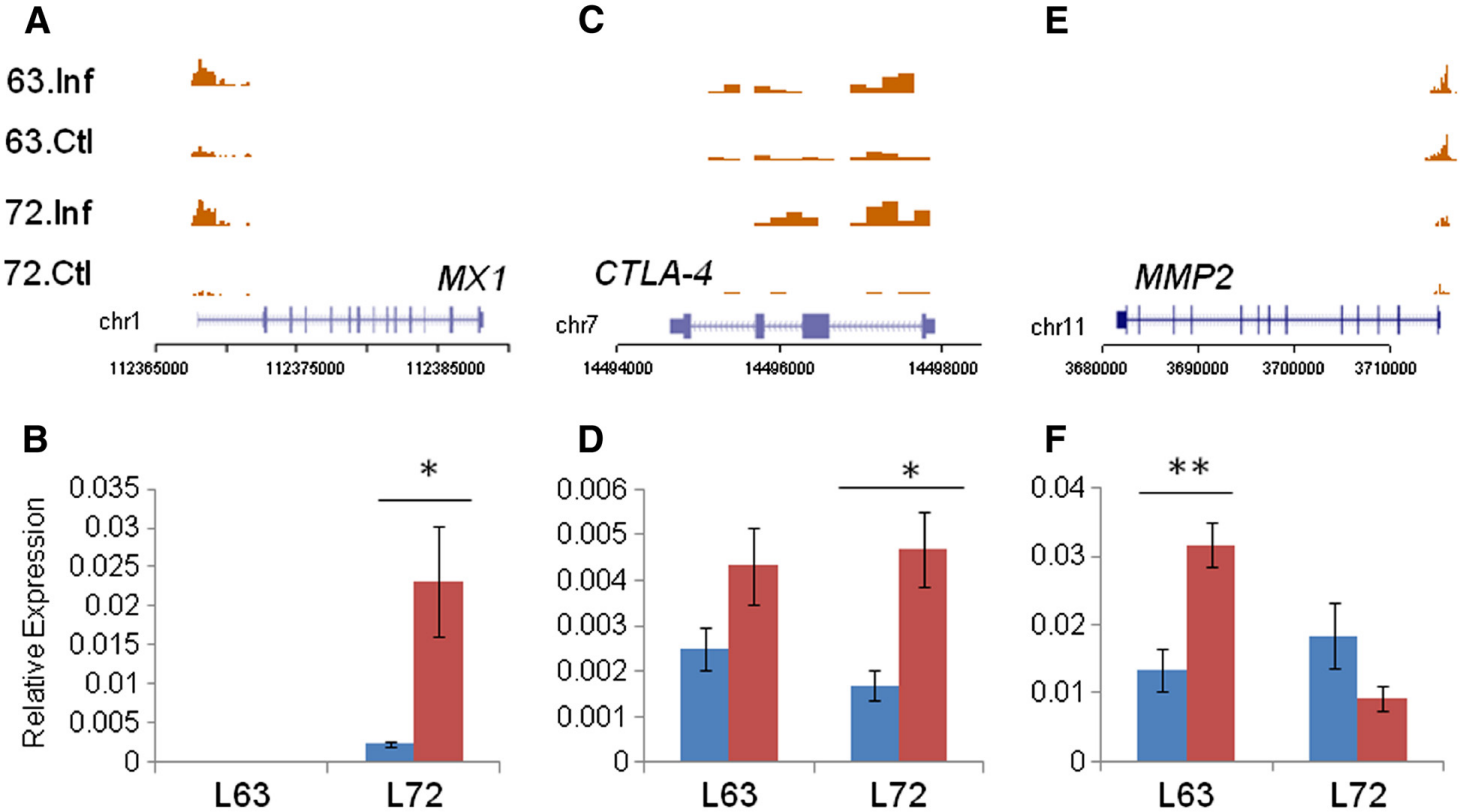
**Genes related to MD show differential H3K4me3 marks.***MX1* (**A**, **B**) and *CTLA-4* (**C**, **D**) show increased H3K4me3 marks and higher expression in infected individuals from both lines; *MMP2* (**E**, **F**) exhibits higher levels of H3K4me3 in susceptible line 7_2_. n = 4; * = significant at p < 0.05; ** = significant at p < 0.01; *** = significant at p < 0.001.

*MX1* is a zinc-finger transcription factor that has antiviral properties against a large number of viruses. *MX1* was upregulated after MDV infection [17] although its contribution to MD progression is unknown. MDV infection induced a highly significant increase in H3K4me3 enrichment in the promoter region of *MX1* in both lines (line 6_3_: p = 1.28x10^-7^, line 7_2_: p = 4.26x10^-9^; Figure 2A). We observed a concurrent increase in transcript levels after MDV infection in line 7_2_ (p = 0.0264; Figure 2B); *MX1* expression in line 6_3_ showed a similar trend (fold change = 38.22, p=0.085) although mRNA levels were much lower.

*CTLA-4* is a cell surface glycoprotein expressed on CD4+ and CD8+ T lymphocytes that is a powerful negative regulator of T-cell activation [18]. The CTLA4 protein is expressed on T lymphocytes soon after activation and regulates T-cell proliferation, production of IL-2 and also supports the function of T_reg_ cells that suppress immune response [19]. Previous studies have reported an increase in *CTLA-4* expression after MDV infection [20]. We found an increase in H3K4me3 enrichment in line 7_2_ as a result of MDV infection (p = 0.0003) and there was a similar trend in line 6_3_ (Figure 2C). Consistent with the above, there was a significant increase in transcript levels after MDV infection in line 7_2_ (p = 0.04) and a small increase in line 6_3_ (Figure 2D).

*MMP2* plays a key role in the degradation of the extra-cellular matrix, and an increase in expression has been associated with increasing tumor cell migration and tumor angiogenesis [21,22]. *MMP2* was upregulated during the neoplastic stage of MD infection in susceptible birds [23] but downregulated in response to MDV infection during early lytic infection in susceptible and resistant chickens [17]. We observed a slight increase in H3K4me3 enrichment after MDV infection in both lines, while line 7_2_ exhibited significantly lower levels than line 6_3_ (p = 0.0016; Figure 2E). This was coupled with increased *MMP2* expression in line 6_3_ after infection (p = 0.0068) while there was no such change in line 7_2_ (Figure 2F).

### Genes related to cancers show epigenetic changes in response to MD

**Figure 3:**
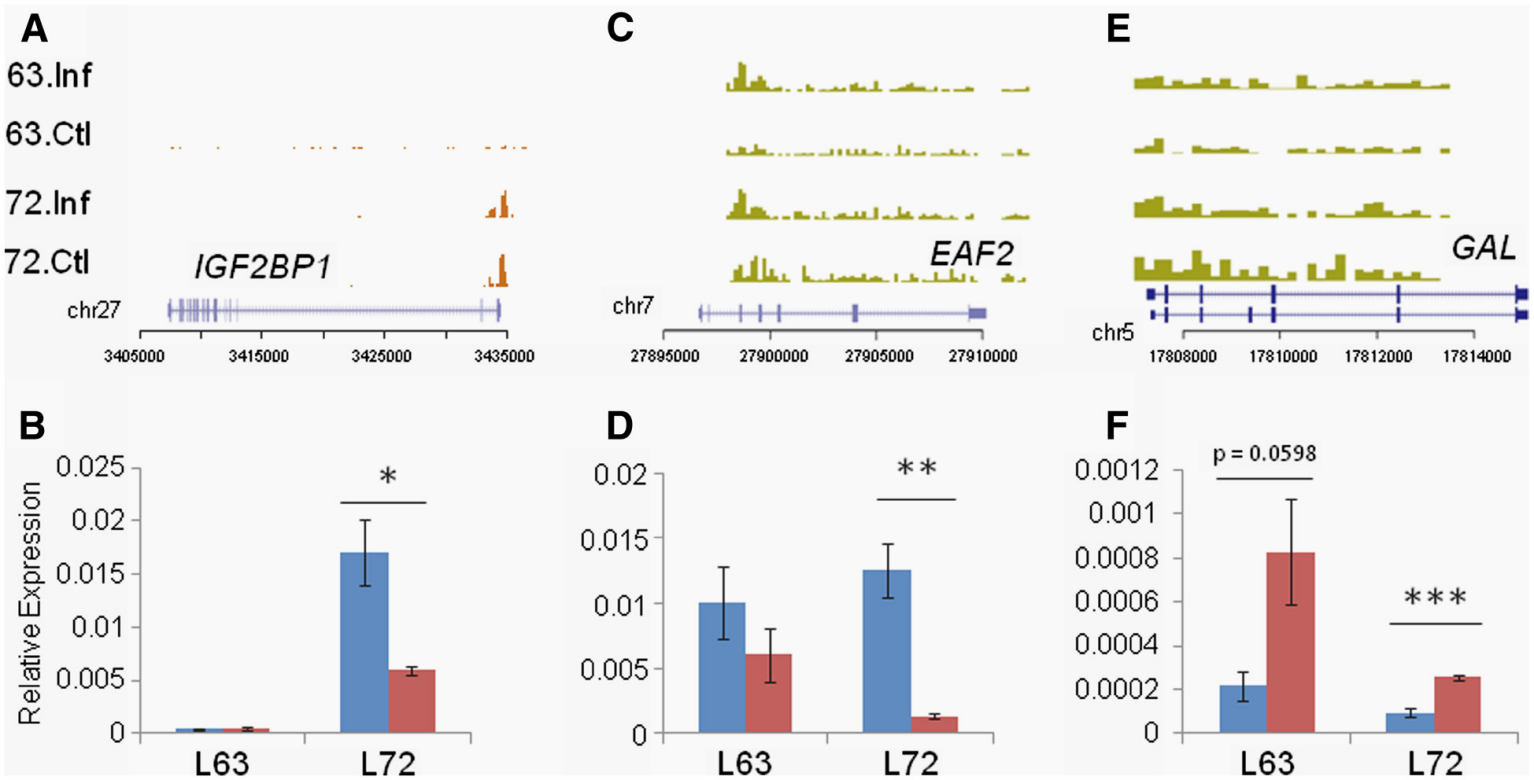
**MD induces epigenetic changes in genes related to various cancers.***IGF2BP1* (**A**, **B**) shows differential H3K4me3 marks and increased expression in susceptible birds while *EAF2* (**C**, **D**) and *GAL* (**E**, **F**) have differential H3K27me3 levels on the gene body. n = 4; * = significant at p < 0.05; ** = significant at p < 0.01; *** = significant at p < 0.001.

We observed differential histone marks on several genes that have been associated with other cancers but not in the context of MDV infection. Insulin-like growth factor 2 binding protein 1 (*IGF2BP1*) is an RNA-binding factor that regulates the translation of mRNAs produced by certain genes like *IGF2* and *ACTB*. Increased expression of *IGF2BP1* has been implicated in the development and progression of cancers of various organs, e.g. lung, brain, breast and colon [24-27]. There was no change in the H3K4me3 enrichment levels induced by MDV infection although a significantly higher level of enrichment was present in line 7_2_ (p = 4.21x10^-13^; Figure 3A). Transcript levels in line 7_2_ were much higher than in line 6_3_, but reduced in response to MDV infection (p = 0.044) (Figure 3B).

ELL associated factor 2 (*EAF2*) is a testosterone regulated apoptosis inducer and tumor suppressor. Inactivation of *EAF2* has been shown to lead to tumors in multiple organs [28]. There was a significant increase in H3K27me3 levels after MDV infection in line 6_3_ (p = 0.0414) while among uninfected chickens these levels were markedly higher in line 7_2_ (p = 0.0138; Figure 3C). However, *EAF2* expression was drastically reduced after MDV infection in line 7_2_ (p=0.0016) but showed only a small decrease in line 6_3_ (Figure 3D).

**Figure 4:**
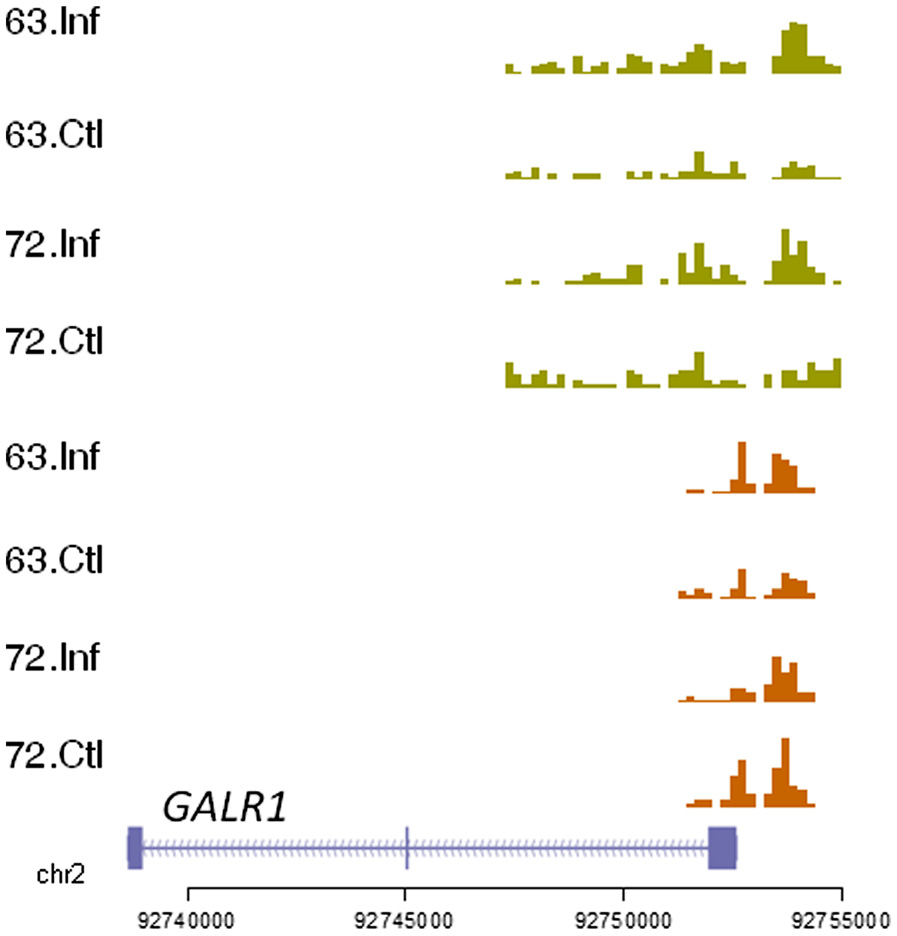
**Significant H3K4me3 and H3K27me3 enrichment around *GALR1.*** The anti-proliferative *GAL* receptor *GALR1* exhibited both active and repressive marks. There is no change in H3K4me3 levels but a definite increase in H3K27me3 levels after infection in line 7_2_.

Galanin (*GAL*) is a neuropeptide that modulates various physiological functions, such as, inhibition of insulin secretion and stimulation of growth hormone secretion. Three galanin receptors are known (*GALR1*, *2* and *3*): the expression of *GALR1* has anti-proliferative effects while *GALR2* can be both anti- or pro-proliferative in function. Therefore, the *GAL* system is considered to be a promising candidate for detection and treatment of various cancers [29,30]. We observed an increase in H3K27me3 levels on *GAL* after infection in both lines (Figure 3E). Also, expression levels were significantly lowered after MDV infection in line 7_2_ (p = 0.00087) while there was a similar trend in line 6_3_ (p = 0.051; Figure 3F). Interestingly, *GALR1* had both H3K4me3 and H3K27me3 enrichments (Figure 4) although *GALR2* showed no significant histone marks (Additional file 1: Figure S6).

### Chromatin co-localization patterns reveal putative bivalent genes

Regions of chromatin having both active and repressive marks are said to be bivalent and have been shown to play important roles in development and genetic imprinting [31,32]. For example, bivalent domains have been shown to mark promoters of genes that are subsequently silenced in tumors by DNA hypermethylation indicating their importance in cancer [33]. A mono-allelic bivalent chromatin domain that controls tissue-specific genomic imprinting at a specific locus was recently found in mice [32]. To investigate the presence of such bivalent chromatin states and the possible effect of MDV infection, we defined bivalent genes as those having H3K4me3 reads (TSS ± 500 bp) greater than 30 reads per million mapped reads (RPM) and H3K27me3 reads (gene body) greater than 2 RPM, respectively (~85^th^ percentile). This filtering process yielded a list of 99 putative bivalent genes (Additional file 4).

Functional annotation clustering of the above genes using DAVID [34,35] revealed significant enrichment of several immune-related functions. These included transcription factor *EGR1* which is reported to have tumor suppressor properties, genes involved in lymphocyte activation and differentiation such as *BCL6*, *CD4* and *SMAD3* and genes *TLR3* and *TIRAP* that are part of the toll-like receptor signaling pathway. Bivalent domains were also present on a variety of transcription factors with immune-related functions such as *CITED2*, a transactivator that regulates NF-κB, *MYC* a transcription factor associated with hematopoetic tumors and *RHOB* a Ras family homolog that mediates apoptosis in tumor cells after DNA damage. Moreover, all genes involved in the top five functional annotation clusters showed higher chromatin levels in line 7_2_ primarily after MDV infection (Additional file 5).

### Bivalent domains are altered in response to MD

We further investigated the effect of MD on bivalent chromatin domains observed on *BCL6*, *CITED2*, *EGR1*, *CD4* and *TLR3* (Additional file 1: Figure 5 and S7). Interestingly, three of these genes, *CITED2, BCL6* and *EGR1*, showed identical epigenetic and transcriptional signatures.

**Figure 5:**
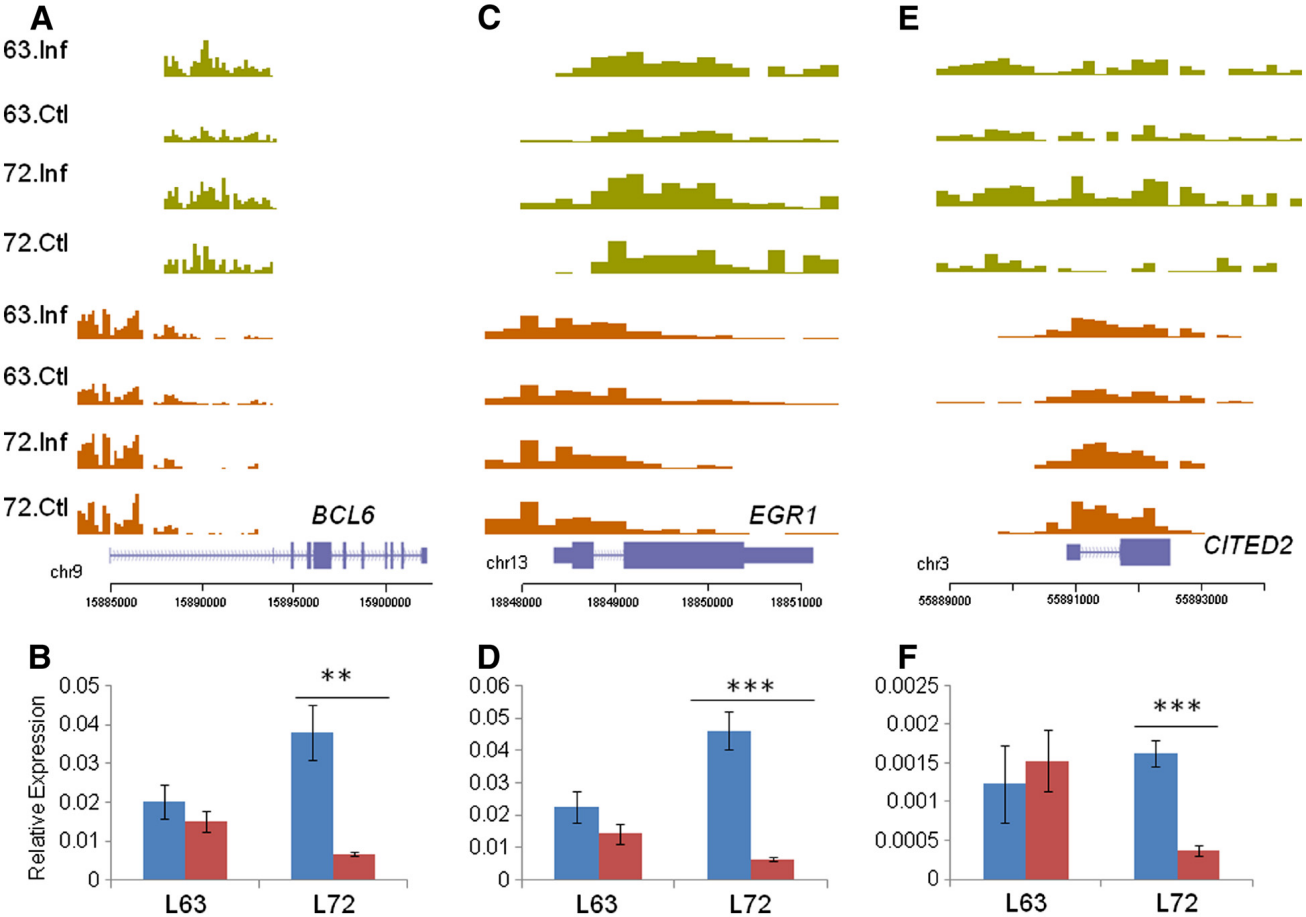
**Bivalent domains on transcriptional regulators are altered by MD.** H3K4me3 (orange) and H3K27me3 (green) profiles and associated transcript levels of *BCL6* (**A**, **B**), *EGR1* (**C**, **D**) and *CITED2* (**E**, **F**). In all three cases we observe a slight increase in H3K27me3 induced by MDV infection in line 7_2_ and a concurrent significant decrease in transcript levels while increase in active and repressive marks appear to cancel each other out in line 6_3_.

*CITED2* is a member of the p300/CBP co-activator family that has intrinsic histone acetyltransferase activity and plays a major role in regulating and coordinating multiple complex cellular signals to determine the expression level of a gene [36]. B-cell CLL/lymphoma 6 (*BCL6*) is a zinc finger protein that functions as a transcriptional repressor which was downregulated at 15 dpi in spleen tissues from F1 progeny (15I_5_ X 7_1_) of MD-susceptible chickens [20]. *EGR1* belongs to a group of early response genes induced by a variety of signaling molecules such as growth factors, hormones and neurotransmitters that is believed to play a major role in cell proliferation and apoptosis [37]. Overexpression of *EGR1* promotes tumor growth in kidney cells [38] but suppresses growth and transformation in other cell types, e.g. fibroblasts and glioblastoma cells [39].

In each of the above genes, both active and repressive chromatin marks were increased in response to infection in line 6_3_ chickens. However, in line 7_2_, there was a definite increase in H3K27me3 marks but no change in H3K4me3 (Figures 5A, C, E). Transcript levels were in agreement with this observation: infected line 7_2_ chickens showed a significant downregulation (*CITED2*: p=0.0004; *BCL6*: p=0.0048; *EGR1*: p=0.0005; Figures 5B, D, F), while there were no such changes in line 6_3_.

On the other hand, *TLR3* and *CD4* showed a slight increase in H3K4me3 marks after MDV infection while there were no appreciable changes in H3K27me3 levels. In keeping with the epigenetic changes, there was a small increase in gene expression in infected birds from both lines (Additional file 1: Figure S7).

## Discussion

Immune parameters that are known to play a major role in genetic resistance to MDV are correlated with innate immune responses, such as NK cell activity, production of nitric oxide and cytokines, such as, interferons. Recent studies have identified host cytokines such as IL-18 and IFN-γ that contribute to the initiation and continuation of latency [40]. However, cytokine levels can undergo rapid flux in response to infection, and consistent with this, we did not observe any epigenetic changes associated with these genes in the MHC-congenic lines used in our study (Additional file 1: Figure S8). This suggests the existence of other extrinsic factors responsible for transcriptional variations between resistant and susceptible chickens at this stage of the disease.

A global comparison of histone modification levels in the two inbred chicken lines yielded some interesting results. As expected, a majority of SERs were found in all the experimental groups, indicating a high level of epigenetic similarity between the lines in addition to inherent genetic similarity. In the case of H3K27me3, the percentage of ubiquitous SERs was relatively low (23.4%), although this was likely due to lower precision of the peak caller for broad chromatin marks. Besides, most of the SERs detected in a subset of samples corresponded to regions of low enrichment, which may also be the reason behind the relatively low number of differential SERs detected in our study.

Genes that have been previously associated with MD and various other cancers showed differential marks that are either MD-induced (*MX1, CTLA-4, EAF2* and *GAL*) or line-specific (*IGF2BP1* and *MMP2*). The increase in H3K4me3 marks around the TSS of *MX1,* a gene with known antiviral properties, appeared to be correlated with upregulated expression in both lines in response to MDV infection. However, lowered overall mRNA levels in the resistant line suggest additional factors could be involved in the regulation of this gene. Similarly, increased mRNA levels of the lymphocyte surface marker *CTLA4* is possibly due to the presence of larger numbers of T cells in MDV infected birds as a result of higher levels of infection. *EAF2* functions as an apoptosis inducer in addition to being a tumor suppressor, and therefore, its downregulation could contribute to higher tumor incidence rates in line 7_2_. However, it is not clear why a significant increase in H3K27me3 levels did not have any effect on transcript levels in the resistant line.

*IGF2BP1* is believed to act by stabilizing the mRNA of the *c-myc* oncogene and therefore, the higher expression of this gene and a more stable c-myc gene product might play a role in increasing MD susceptibility in line 7_2_ birds via increased cell proliferation and transformation. The matrix metalloprotease *MMP2* is upregulated after infection in the resistant line 6_3_, similar to the previously observed increase at the neoplastic stage of MD. However, mRNA levels were similar in the two lines before infection contrary to the difference in H3K4me3 levels suggesting that additional factors are responsible for regulating this gene.

The correlation between observed differential histone marks and transcript levels was moderate at best. Indeed, differential H3K4me3 marks were strongly predictive of gene expression levels but the correlation between H3K27me3 and mRNA levels was relatively poor. There could be various reasons for this – indeed, H3K27me3 levels had a non-linear relationship with gene expression with higher marks showing little difference in the effect on expression. Therefore, in this tissue, the levels of H3K27me3 may not be a very good indicator of gene expression levels. Also, the transcription of these genes might be controlled by other factors with the change in H3K27me3 levels only incidental.

Bivalent domains were detected on transcriptional regulators *BCL6*, *CITED2* and *EGR1* and the galanin receptor *GALR1.* The epigenetic and transcriptional signatures observed on these genes indicated that they were poised at the latent stage of the disease, but with crucial differences in the two lines. Increased repressive marks in the susceptible line correlated with significant downregulation of the genes, while in line 6_3_, the increase in both marks appeared to compensate for each other with no eventual effect on gene transcription. This suggests that some ‘poised’ bivalent genes can become highly repressed even with a relatively small increase in H3K27me3 marks. The change in the chromatin levels could also be correlated with an increase in cell populations having the repressive mark. Taken together, the above findings point towards the existence of finely balanced epigenetic control of transcription, which may be necessary to mount a rapid response by the immune system. However, this machinery could potentially be hijacked by a pathogen and result in an aberrant phenotype. The effect of MDV infection on the bivalent domain on *GALR1*, in particular, suggests the repression and potential loss of its anti-proliferative effects. Thus, the galanin system possibly plays an important and hitherto unknown role in MD progression and could be a novel target for long-term control of the disease.

One of the major players in MDV-induced malignant transformation is Meq, a virus-encoded oncoprotein that has diverse functions, e.g. transactivation, chromatin remodeling and regulation of transcription. Meq interacts with and sequesters the tumor suppressor protein p53, resulting in the dysregulation of cell-cycle control [7] and inhibition of the transcriptional and apoptotic activities of the protein [41]. Several genes that show epigenetic changes in response to MDV infection have been associated with p53. Downregulation of *CITED2* stabilizes the p53 protein leading to its accumulation [42]. The *BCL6* gene product is believed to contribute to lymphomagenesis by inactivation of p53 [43]. Besides, *EAF2* has also been shown to interact with and suppress the function of p53 [28]. The downregulation of all of the above genes in susceptible birds after MDV infection points towards the increased production of p53 and a robust anti-tumor response. That we still observe higher tumor incidence rates in this line, suggests the presence of large amounts of inactivating viral Meq protein which, in turn, indicates that increased numbers of MD-infected cells are present in the susceptible line at this stage of the disease. A majority of tumors are believed to result from the viral transformation of CD4+ T cells some of which are infected at the latent stage of MD [44]. The larger number of virus-infected cells produced in the susceptible line is possibly due to lowered immunocompetence as a result of the early stages of infection. Thus, a more detailed investigation of the early cytolytic stage of MD is necessary to shed further light on the causes behind the divergent response to MD observed in these birds.

Whole tissues represent a mixture of various cell populations, and observed epigenetic changes might be due to a change in chromatin marks in a particular cell type or a variation in the relative number of cells carrying active or repressive histone marks. However, such *in vivo* studies are representative of the host response at a systems level wherein different cell types might interact in a cooperative manner to fight infection. Thus, while the study of pure cell populations is likely to yield greater discriminative power, the investigation of tissue macroenvironments is, perhaps, closer to reality.

This study focused on the thymus tissue as it is a major immune organ and contains a significant population of T lymphocytes in various stages of differentiation. Our earlier study of the MDV-induced transcriptome in these birds indicated the presence of line-specific differences at the latent stage of MD [45]. In addition, birds susceptible to MD suffer thymic atrophy during the early stages of infection [46], indicating the importance of understanding changes in this organ to elucidate the mechanisms involved in disease progression. Ongoing studies in our lab include other tissues, e.g. spleen [47], and a time-course through the various stages of infection, to further investigate the systemic effects of MD and the epigenetic basis of MD resistance.

## Conclusions

We studied the effect of latent MDV infection on two chromatin modifications in inbred chicken lines exhibiting different degrees of resistance to MD. Several genes showed changes in histone enrichment and this response was often significantly different between the two chicken lines. A detailed analysis of co-localization patterns of the chromatin marks revealed the presence of bivalent domains on a number of immune-related transcriptional regulators. More importantly, these domains showed marked changes in response to MDV infection and provide further evidence of the far-reaching epigenetic effects of MD. Our results suggest putative roles for the *GAL* system in MD progression. A majority of the differential chromatin marks are also suggestive of increased levels of viral infection in the susceptible line symptomatic of lowered immunocompetence in these birds at early stages of the disease. In summary, our study provides further insight into the mechanisms of MD progression while revealing a complex landscape of epigenetic regulatory mechanisms. Further work is necessary to fully elucidate the underlying mechanisms of MD, but our results suggest that this is a promising step towards a deeper understanding of this complex disease.

## Methods

### Animals and viruses

Two specific-pathogen-free inbred lines of White Leghorn either resistant (6_3_) or susceptible (7_2_) to MD were hatched, reared and maintained in Avian Disease and Oncology Laboratory (ADOL, Michigan, USDA). Four chickens from each line were injected intra-abdominally with a partially attenuated very virulent plus strain of MDV (648A passage 40) at 5 days after hatch with a viral dosage of 500 plaque-forming units (PFU). Infected and control chickens from both lines (n = 4) were terminated at 10dpi to collect thymus tissues. All procedures followed the standard animal ethics and use guidelines of ADOL.

### Quantification of MDV loads in thymus

The MDV gene *ICP4* was used for quantification of viral genomic DNA in thymus as previously described [48]. Quantitative PCR was performed by using 100 ng/μl of genomic DNA on the iCycler iQ PCR system (Bio-Rad, USA) and QuantiTect SYBR Green PCR Kit (Qiagen, USA) (Additional file 1: Figure S9). The relative MDV loads were determined by normalizing to a single-copy gene *Vim* (vimentin) [49]. The primers for *Vim* are as follows: Forward: 5’-CAGCCACAGAGTAGGGTAGTC-3’; Reverse: 5’-GAATAGGGAAGAACAGGAAAT-3’.

### Chromatin Immunoprecipitation and Illumina Sequencing

Chromatin immunoprecipitation (ChIP) was carried out using thymus samples from MDV infected and controls birds [50]. About 30 mg thymus samples from three individuals were cut into small pieces (1 mm^3^) and digested with MNase to obtain mononucleosomes. PNK and Klenow enzymes (NBE, Ipswich, MA, USA) were used to repair the ChIP DNA ends pulled down by the specific antibody. A 3′ adenine was then added using Taq polymerase and a pair of Solexa adaptors (Illumina, USA) ligated to the repaired ends. Seventeen cycles of PCR was performed on ChIP DNA using the adaptor primers and fragments with a length of about 190 bp (mononucleosome + adaptors) were isolated from agarose gel. Subsequently, cluster generation and ChIP-sequencing (ChIP-Seq) using the purified DNA was performed on the Solexa 1G Genome Analyzer (Illumina, USA) following manufacturer protocols. The antibodies used and the total number of reads obtained for each sample is listed in Additional file 6.

### Read mapping and summary counts

Sequence reads obtained from the Illumina 1G Genome Analyzer were aligned to the May 2006 version of the chicken genome (galGal3) using Maq version 0.7.1 [51]. Default alignment policies of Maq were enforced: a valid alignment could have a maximum of two mismatches and if a read aligned equally well to multiple places in the genome, one was chosen at random. If multiple reads mapped to the same genomic location only one was kept to avoid amplification bias. Summary read counts were calculated using non-overlapping windows of 200 bp for visualization and normalized to per million mapped reads in each sample for the purpose of comparisons.

### Identification of enriched regions

Summarized read counts were subjected to peak calling with SICER [52]. The source code was modified to include support for the chicken genome. Fragment length was specified to be 190. A window size of 200 bp and gap size of 400 bp was used for the analysis. The E-value for estimating significant peaks was set to 100. For the purposes of comparing different samples, SERs found in similar genomic regions of different samples were merged to obtain a consolidated list as follows: SERs from one sample were used to initialize the list. For each such region *M,* we searched for overlapping SERs in the next sample. In the case of an overlap between *M* and a significant region, *S*, the merged region was updated to include both *M* and *S*. This procedure was iterated over all samples to obtain a consolidated list of merged SERs.

### Gene annotation and genomic distribution of SERs

RefSeq and Ensembl gene annotations were downloaded from UCSC genome browser [53]. As there were only 4306 RefSeq genes in the database, we included Ensembl genes in our analysis to improve genome-wide coverage. There were 17858 annotated genes in the Ensembl database, which include validated and predicted genes. Redundancies between the databases were listed and accounted for, yielding a non-redundant list of 18198 genes with 4306 RefSeq genes and 13892 Ensembl genes. We then searched for overlaps between merged SERs and the non-redundant list of annotated genes. For H3K4me3, an SER was annotated with a gene if it overlapped the TSS region of the gene whereas in the case of H3K27me3, a valid overlap constituted an SER overlapping the gene body. To calculate the genomic distribution we counted all SERs having an overlap with one of the following regions: promoter (TSS ± 1 kb), exons, introns, 5’ UTR and 3’ UTR.

### Histone modification profiles and differential chromatin marks

Genes were divided into 10 sets based on their absolute expression and representative sets corresponding to high, medium, low and no expression were chosen for visualization. We defined the gene body as the region between the transcription start site (TSS) and the transcription termination site (TTS). Histone modification profiles surrounding the gene body were calculated in 3 distinct regions: 5000 bp upstream of the 5’ end, gene body and 5000 bp downstream of the 3’ end of the gene. For reads falling within the gene body, read counts were obtained in bins 5% of the gene length while 1000 bp windows were used for the 5’ and 3’ flanking regions. The read counts in all cases were normalized to the total number of genes in the categories and total number of reads in the sample. We also compared gene expression to histone modification levels by plotting normalized microarray data (Zhang, H. unpublished data) against reads mapping to (i) TSS ± 500 bp and (ii) the gene body for each gene.

Reads mapping to merged SERs were tested for epigenetic changes induced by MDV infection in lines 6_3_ and 7_2_ using DESeq [54]. We used the method ‘blind’ for variance estimation and p-values were corrected for multiple testing using the Benjamini-Hochberg FDR procedure [55]. Statistical significance was defined using a false discovery rate (FDR) threshold of 0.4.

### Validation of ChIP, ChIP-Seq and gene transcription by Q-PCR

Quantitative real-time RT-PCR was used to validate the quality of the ChIP and gene transcript levels on the iCycler iQ PCR system (Bio-Rad, Hercules, CA, USA). The real-time RT-PCR reactions were performed with a QuantiTect SYBR Green PCR Kit (Qiagen, Valencia, CA, USA) according to the manufacturer’s instructions. An online primer system ( http://frodo.wi.mit.edu/primer3/) was used to design the primers and four biological and four technical replicates were performed. The primer sequences are shown in Additional file 7.

### Data access

Raw and processed ChIP-Seq data discussed in this manuscript were deposited in the NCBI Gene Expression Omnibus (GEO) ( http://www.ncbi.nlm.nih.gov/geo/) under Series accession number GSE24017.

## Competing interests

The authors declare that they have no competing interests.

## Author contributions

AM performed the data analysis and wrote the manuscript. JL performed the ChIP experiments, sequence library preparation, validation of ChIP-Seq results and revised the manuscript. HMZ collected samples and revised the manuscript. JZS designed the experiments and revised the manuscript. All authors read and approved the final manuscript.

## Supplementary Material

Additional file 1 Figures S1 to S9. Supplementary Figures S1 to S9. Click here for file

Additional file 2 **Differential H3K4me3 marks. Genome-wide differential H3K4me3 marks produced by DESeq (FDR < 0.4) and the associated genes.** P-values from three contrasts are displayed as follows: 63: 63I vs 63N, 72: 72I vs 72N, 63v72N: 63N vs 72N. 63I: line 6_3_ infected, 63N: line 6_3_ control, 72I: line 7_2_ infected, 72N: line 7_2_ control. Click here for file

Additional file 3 **Differential H3K27me3 marks.Genome-wide differential H3K27me3 marks produced by DESeq (FDR < 0.4) and the associated genes.** P-values from three contrasts are displayed as follows: 63: 63I vs 63N, 72: 72I vs 72N, 63v72N: 63N vs 72N. 63I: line 6_3_ infected, 63N: line 6_3_ control, 72I: line 7_2_ infected, 72N: line 7_2_ control. Click here for file

Additional file 4 Putative bivalent genes from colocalization analysis of H3K4me3 and H3K27me3. Click here for file

Additional file 5 Functional annotation clustering of bivalent genes using DAVID. Click here for file

Additional file 6 Sequencing results showing raw and mapped reads for each sample. Click here for file

Additional file 7 Primers used for quantitative PCR validation. Click here for file
