# Supplementary material for: Marek’s disease virus infection induces widespread differential chromatin marks in inbred chicken lines
Source: BMC Genomics. 2012 Oct 16;13:557. doi: 10.1186/1471-2164-13-557 (PMC3505159; doi:10.1186/1471-2164-13-557)
Supplement: Additional file 1 — Figures S1 to S9. Supplementary Figures S1 to S9. [file 1471-2164-13-557-S1.pdf]

Supplementary Figures

Marek's Disease Virus Infection Induces Widespread Differential Chromatin Marks in inbred chicken lines

Apratim Mitra[1], Juan Luo[1], Huanming Zhang[2], Keji Zhao[3]& Jiuzhou Song[1#]

[1]Department of Animal & Avian Sciences, University of Maryland, College Park, MD 20742, USA.
[2]USDA, ARS, Avian Disease and Oncology Laboratory, East Lansing, MI 48823, USA.
[3]Laboratory of Molecular Immunology, National Heart, Lung and Blood Institute, National Institutes of Health, Bethesda, MD 20892, USA.

[#]Corresponding author: songj88@umd.edu

Figure S1: Peak length distributions in different classes of SERs. Probability densities of peak lengths for ubiquitous, line-specific and condition specific SERs. Line-specific and condition-specific SERs predominantly correspond to low enrichment regions for both H3K4me3 (a-d) and H3K27me3 (e-h). 63_inf: line 6_3 infected, 63_non: line 6_3 control, 72_inf: line 7_2 infected, 72_non: line 7_2 control.

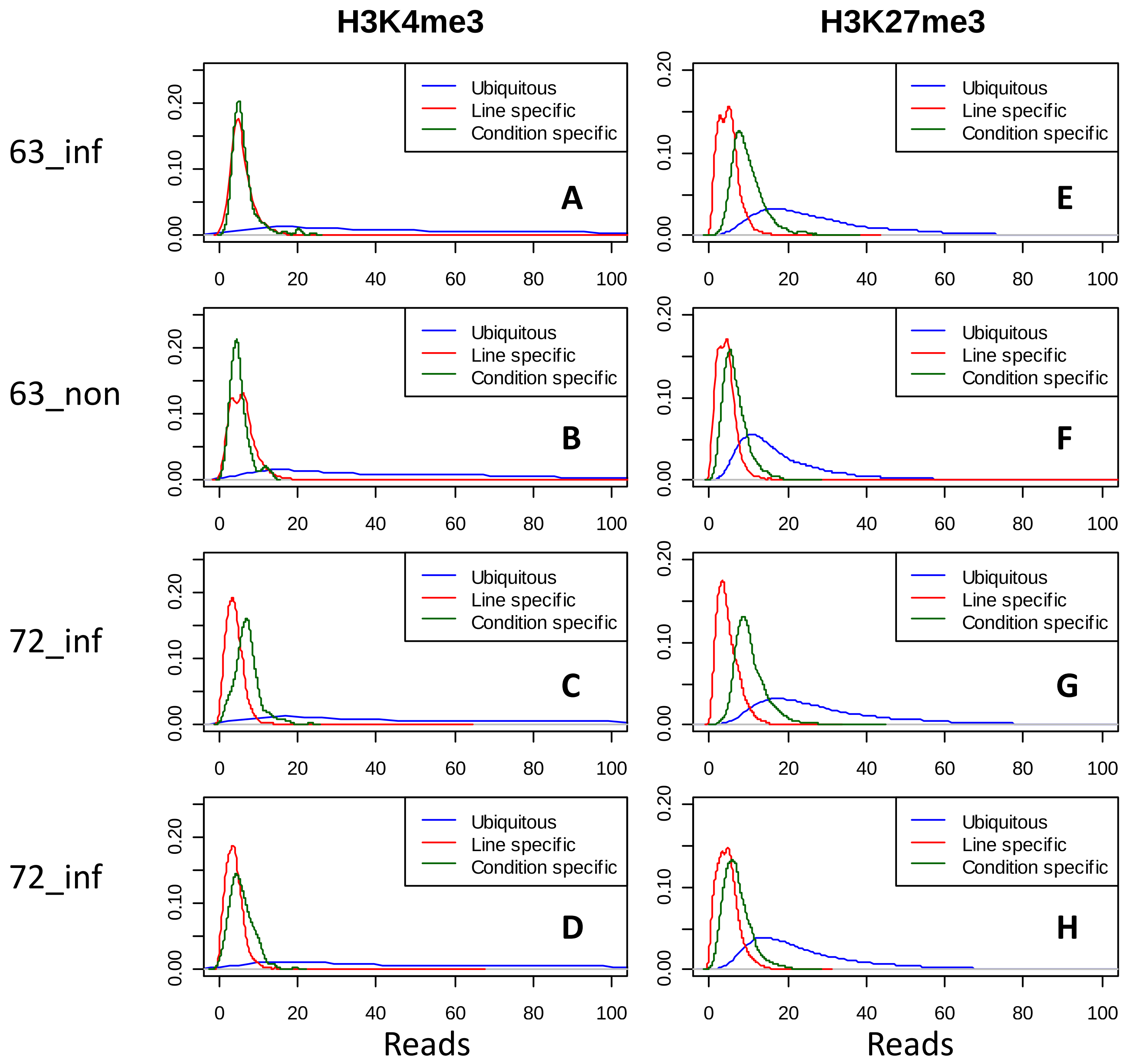

H3K4me3
H3K27me3
63_inf
63_non
72_inf
72_inf
Ubiquitous
Line specific
Condition specific
A
B
C
D
E
F
G
H
0.00
0.10
0.20
0
20
40
60
80
100
Reads
Reads

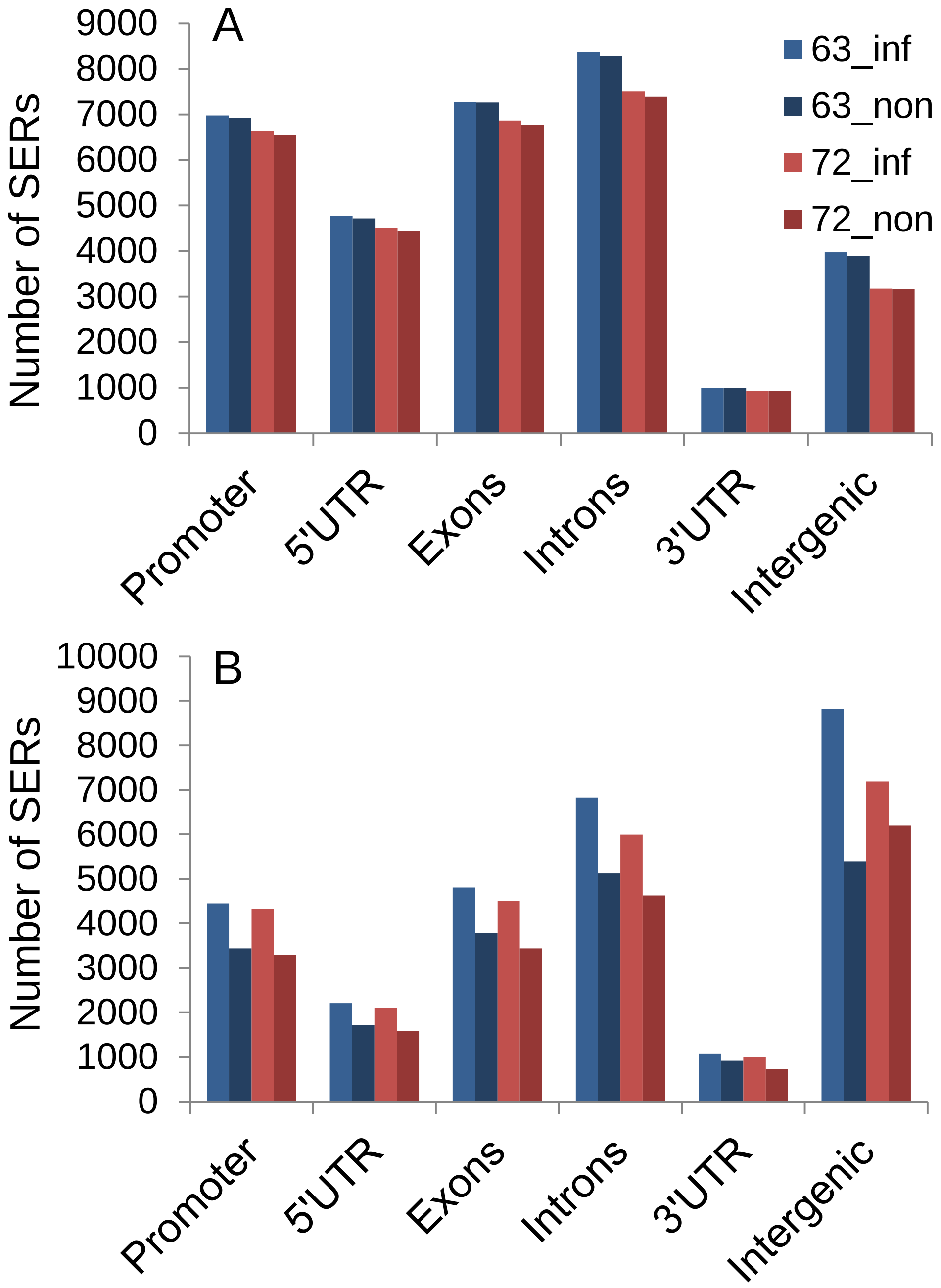

Figure S2. Distribution of SERs over different genomic elements
(a) H3K4me3 and (b) H3K27me3 SERs separated by samples. 63_inf: line 6_3 infected, 63_non: line 6_3 control, 72_inf: line 7_2 infected, 72_non: line 7_2 control.

Figures S3-5: Relationship between gene expression and histone marks. Plots of histone modifications around the gene body (a & b) in genes having high, medium, low and no activity. We also compared epigenetic marks with transcriptional levels: H3K4me3 shows positive correlation with gene expression levels (c) while H3K27me3 exhibits a negative relationship (d). **S3**: Line 6_3 control, **S4**: Line 7_2 control, **S5**: Line 7_2 infected.

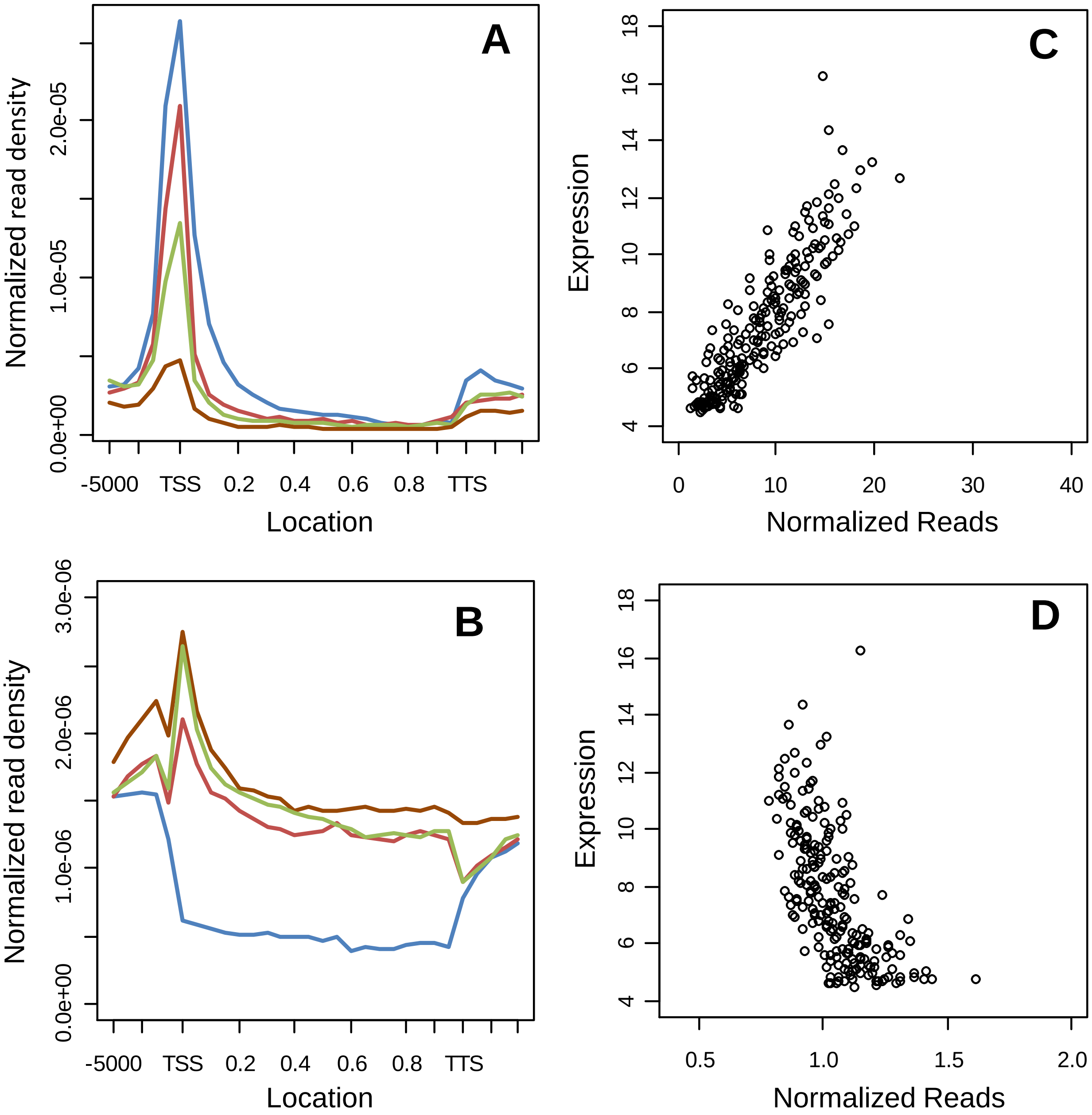

A
Normalized read density
0.0e+00
1.0e-05
2.0e-05
-5000
TSS
0.2
0.4
0.6
0.8
TTS
Location
C
Expression
4
6
8
10
12
14
16
18
0
10
20
30
40
Normalized Reads
B
Normalized read density
0.0e+00
1.0e-06
2.0e-06
3.0e-06
-5000
TSS
0.2
0.4
0.6
0.8
TTS
Location
D
Expression
4
6
8
10
12
14
16
18
0.5
1.0
1.5
2.0
Normalized Reads

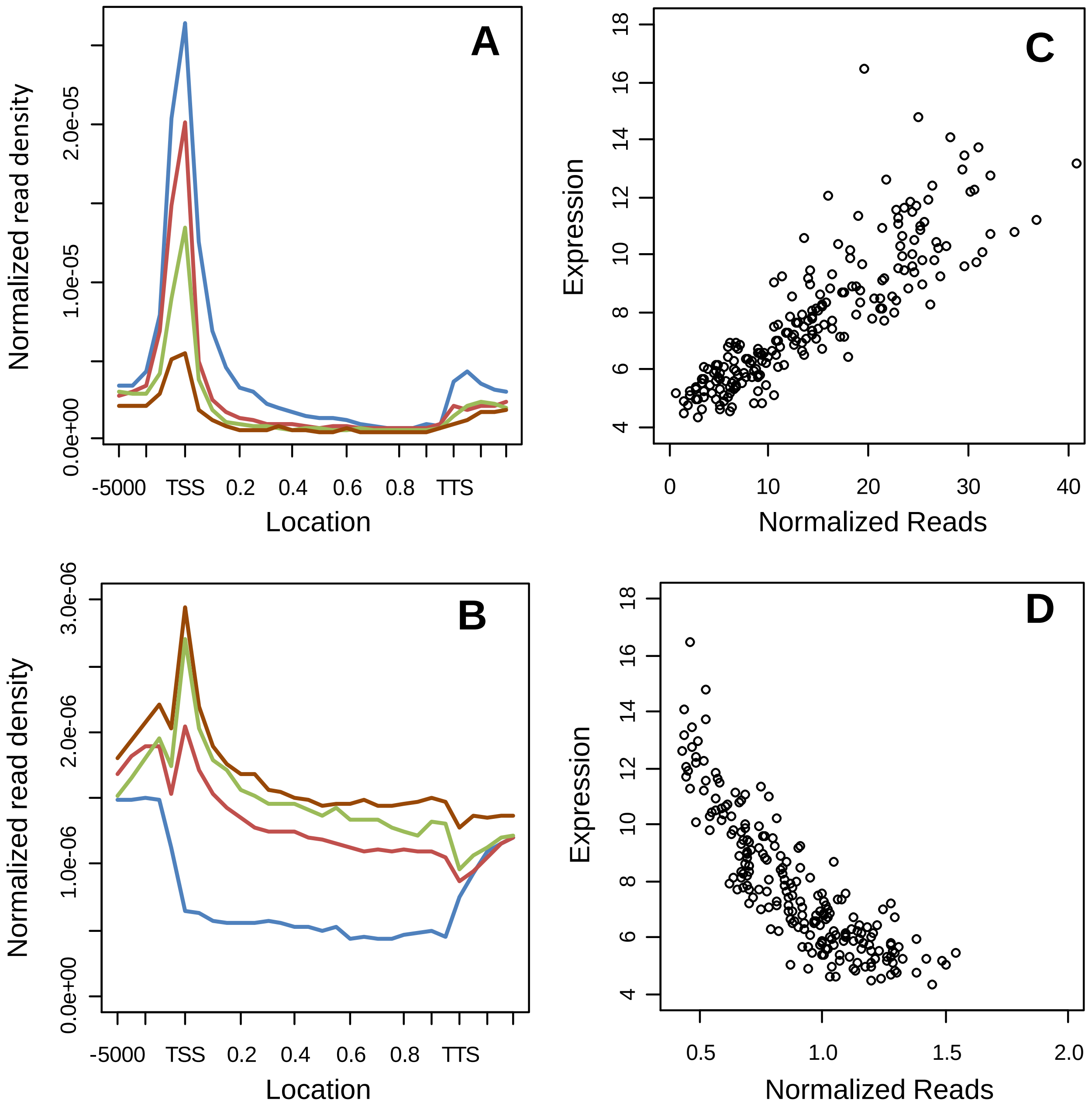

A
B
C
D
Normalized read density
Location
-5000
TSS
0.2
0.4
0.6
0.8
TTS
0.0e+00
1.0e-05
2.0e-05
1.0e-06
2.0e-06
3.0e-06
Expression
Normalized Reads
4
6
8
10
12
14
16
18
0
10
20
30
40
0.5
1.0
1.5
2.0

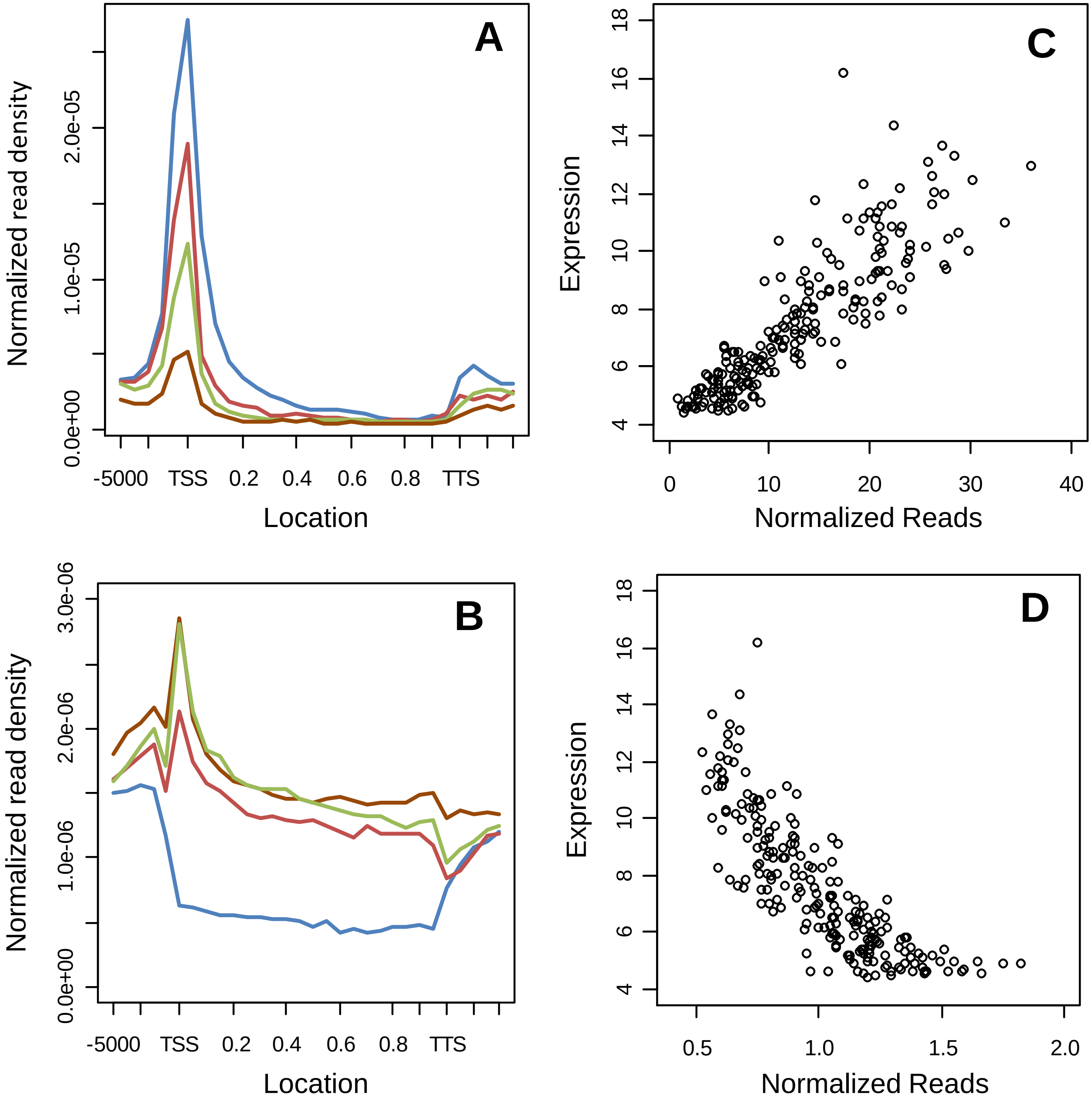
A
Normalized read density
0.0e+00
1.0e-05
2.0e-05
-5000
TSS
0.2
0.4
0.6
0.8
TTS
Location
C
Expression
4
6
8
10
12
14
16
18
0
10
20
30
40
Normalized Reads
B
Normalized read density
0.0e+00
1.0e-06
2.0e-06
3.0e-06
-5000
TSS
0.2
0.4
0.6
0.8
TTS
Location
D
Expression
4
6
8
10
12
14
16
18
0.5
1.0
1.5
2.0
Normalized Reads

Figure S6: H3K4me3 and H3K27me3 marks around *GALR2*. No significant histone marks are observed on *GALR2*

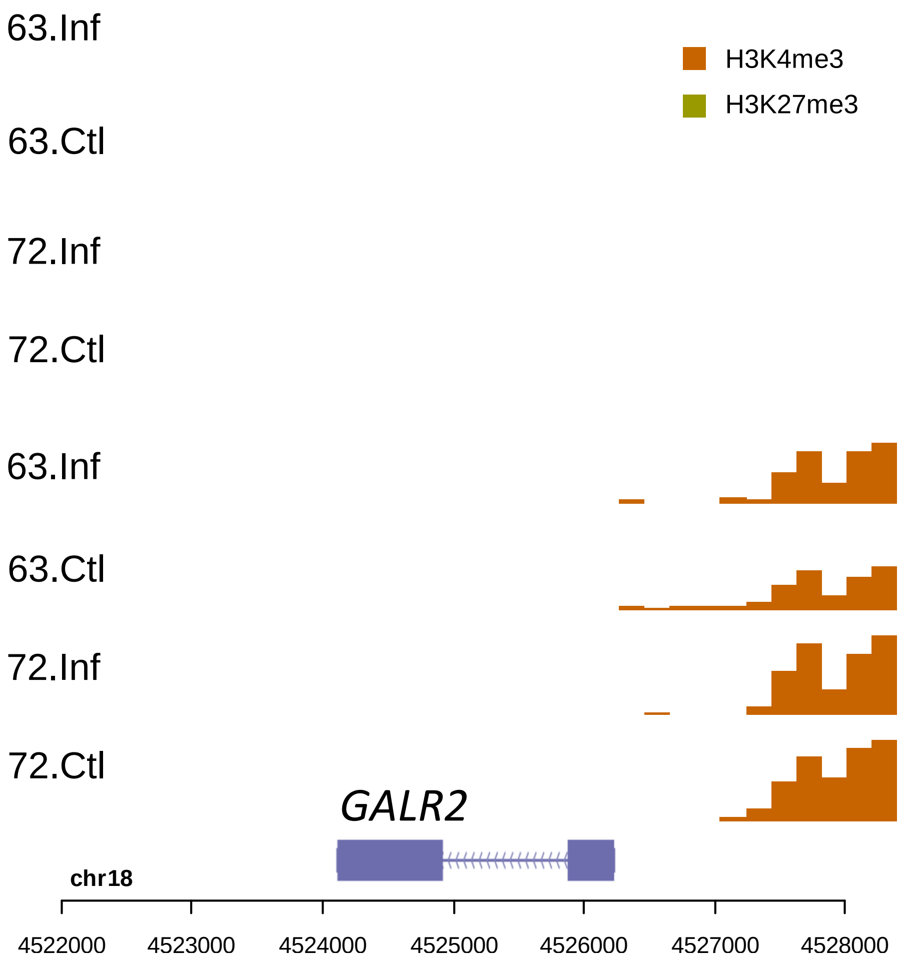

Figure S7: Bivalent domains on some genes are unaffected by virus infection. MDV infection has no effect on the bivalent domains or transcription levels of CD4 and TLR3.

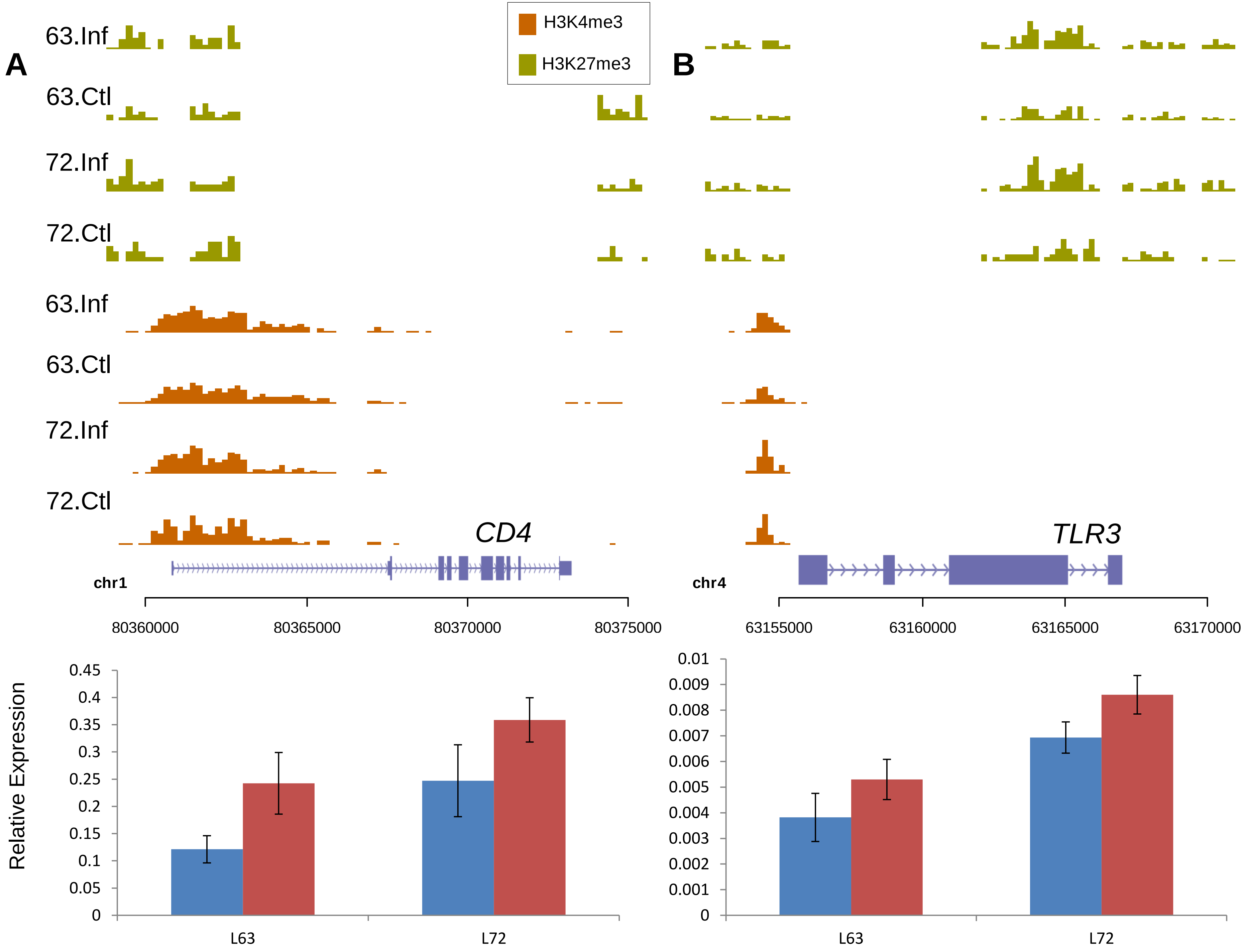
H3K4me3
H3K27me3
A
B
63.Inf
63.Ctl
72.Inf
72.Ctl
63.Inf
63.Ctl
72.Inf
72.Ctl
CD4
TLR3
chr1
chr4
80360000
80365000
80370000
80375000
63155000
63160000
63165000
63170000
Relative Expression
0.45
0.4
0.35
0.3
0.25
0.2
0.15
0.1
0.05
0
L63
L72
0.01
0.009
0.008
0.007
0.006
0.005
0.004
0.003
0.002
0.001
0
L63
L72

Figure S8: Epigenetic profiles of host cytokines (a) IL-18 and (b) IFN-γ. IL-18 does not show any notable changes in response to MDV infection while IFN-γ does not show any SER.

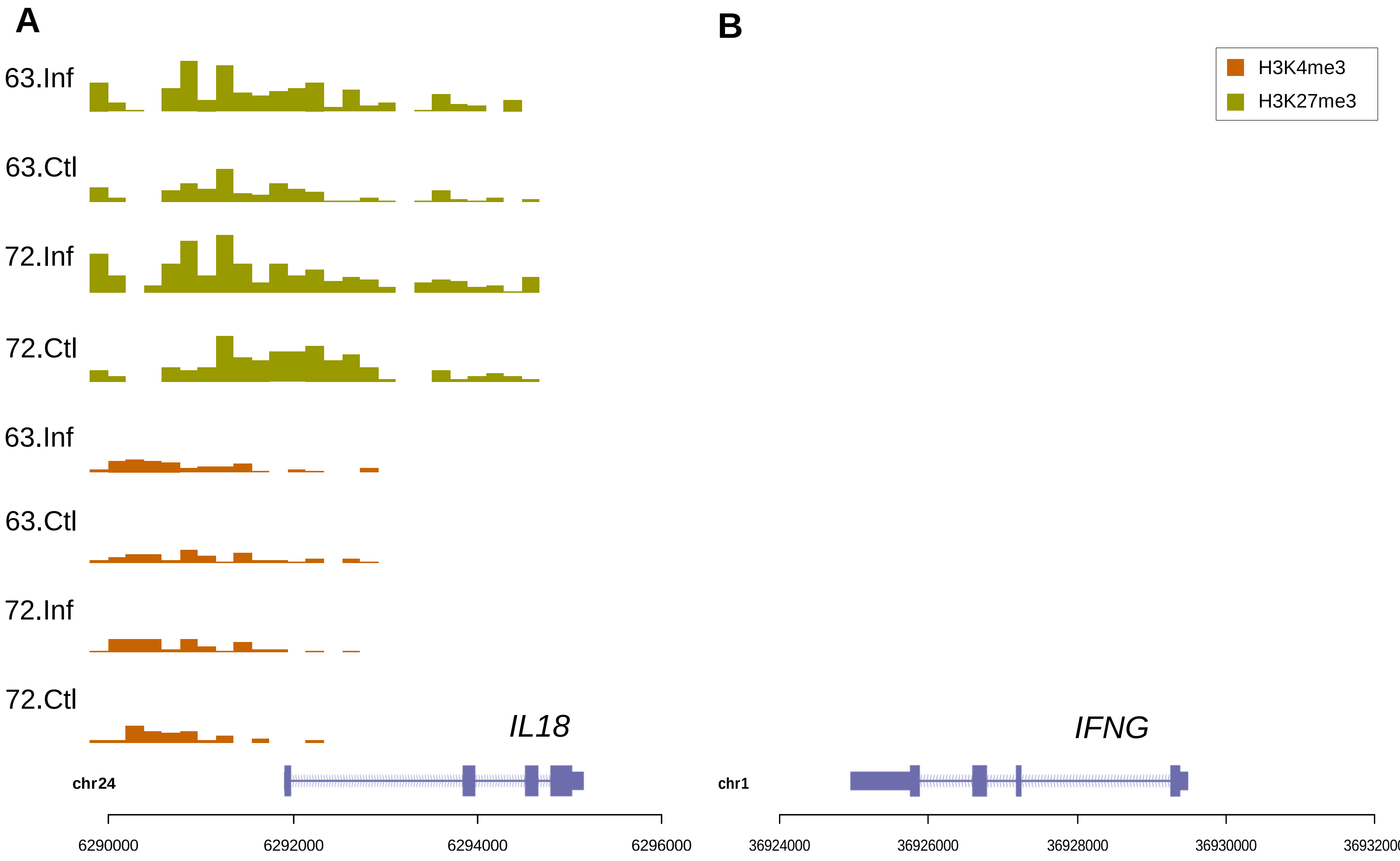

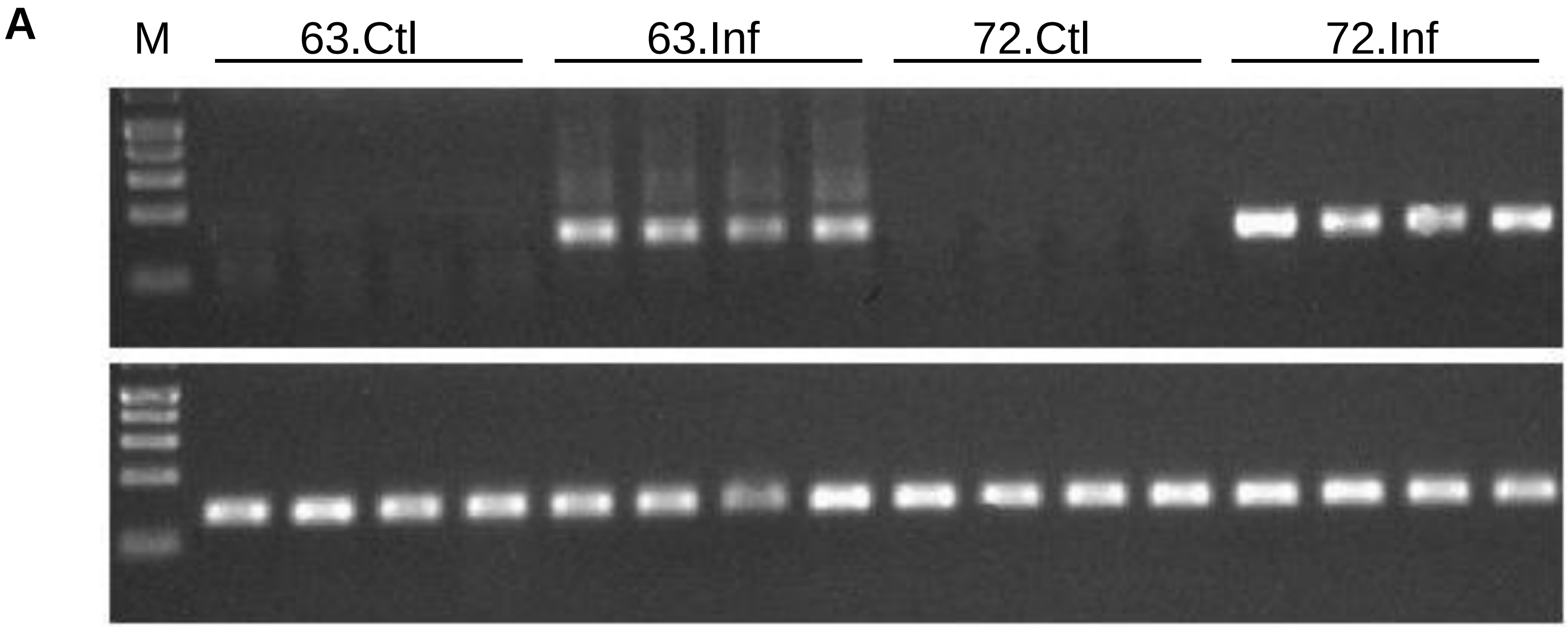

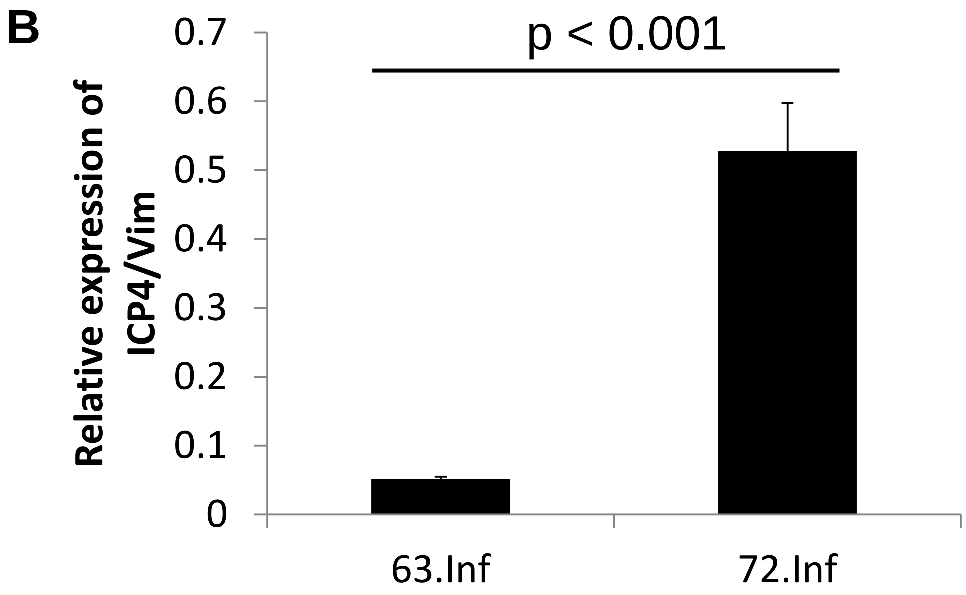

Figure S9: Quantification of virus loads in the MDV-challenge experiment using quantitative RT-PCR. The relative virus load is calculated by quantifying viral *ICP4* normalized to the single-copy *Vim* gene (mean ± SEM, n = 4). (a) Only infected birds from the two lines exhibit measurable virus loads, with (b) the susceptible line 7_2 having a significantly higher number of virus particles ($p < 0.001$).
